# Sieve-based relation extraction of gene regulatory networks from biological literature

**DOI:** 10.1186/1471-2105-16-S16-S1

**Published:** 2015-10-30

**Authors:** Slavko Žitnik, Marinka Žitnik, Blaž Zupan, Marko Bajec

**Affiliations:** 1 Faculty of Computer and Information Science, University of Ljubljana, Večna pot 113, SI-1000 Ljubljana, Slovenia; 2 Department of Molecular and Human Genetics, Baylor College of Medicine, One Baylor Plaza, Houston, TX, 77030, USA; 3 Optilab d.o.o., Župančičeva 8, SI-5270 Ajdovščina, Slovenia

**Keywords:** relation extraction, conditional random fields, gene regulatory networks, literature mining

## Abstract

**Background:**

Relation extraction is an essential procedure in literature mining. It focuses on extracting semantic relations between parts of text, called mentions. Biomedical literature includes an enormous amount of textual descriptions of biological entities, their interactions and results of related experiments. To extract them in an explicit, computer readable format, these relations were at first extracted manually from databases. Manual curation was later replaced with automatic or semi-automatic tools with natural language processing capabilities. The current challenge is the development of information extraction procedures that can directly infer more complex relational structures, such as gene regulatory networks.

**Results:**

We develop a computational approach for extraction of gene regulatory networks from textual data. Our method is designed as a sieve-based system and uses linear-chain conditional random fields and rules for relation extraction. With this method we successfully extracted the sporulation gene regulation network in the bacterium *Bacillus subtilis *for the information extraction challenge at the BioNLP 2013 conference. To enable extraction of distant relations using first-order models, we transform the data into skip-mention sequences. We infer multiple models, each of which is able to extract different relationship types. Following the shared task, we conducted additional analysis using different system settings that resulted in reducing the reconstruction error of bacterial sporulation network from 0.73 to 0.68, measured as the slot error rate between the predicted and the reference network. We observe that all relation extraction sieves contribute to the predictive performance of the proposed approach. Also, features constructed by considering mention words and their prefixes and suffixes are the most important features for higher accuracy of extraction. Analysis of distances between different mention types in the text shows that our choice of transforming data into skip-mention sequences is appropriate for detecting relations between distant mentions.

**Conclusions:**

Linear-chain conditional random fields, along with appropriate data transformations, can be efficiently used to extract relations. The sieve-based architecture simplifies the system as new sieves can be easily added or removed and each sieve can utilize the results of previous ones. Furthermore, sieves with conditional random fields can be trained on arbitrary text data and hence are applicable to broad range of relation extraction tasks and data domains.

## Background

We are witnessing an unprecedented increase in the number of biomedical abstracts, experimental results and phenotype and gene descriptions being deposited to publicly available databases, such as NCBI's PubMed. Collectively, this content represents potential new discoveries that could be inferred with appropriately designed natural language processing approaches. Identification of topics that appear in biomedical research literature was among first computational approaches to predict associations between diseases and genes and has become indispensable to both researchers in the biomedical field and curators [1-4]. Information from publication repositories is often mined together with other data sources. Databases that store relations from integrative mining are for example the OMIM database on human genes and genetic phenotypes [5], the GeneRIF function annotation database [6], the Gene Ontology [7] and clinical drug information from the DailyMed database [8]. Biomedical mining of literature is a compelling way to identify possible candidate genes through integration of existing data.

A dedicated set of computational techniques is required to infer structured relations from plain textual information stored in large literature databases [9]. Relation extraction tools [10] can identify semantic relations between entities found in text. Early relationship extraction systems relied mostly on manually defined rules to extract a limited number of relationship types [11]. Later, machine learning-based methods were introduced to address the extraction task by inferring prediction models from sets of labeled relationship types [12-14]. When no labeled data were available, unsupervised systems were developed to extract relationship descriptors based on the language syntax [10]. Current state-of-the-art systems combine both machine learning and rule-based approaches to extract relevant information from narrative summaries and represent it in a structured form [15,16].

**Figure 1:**
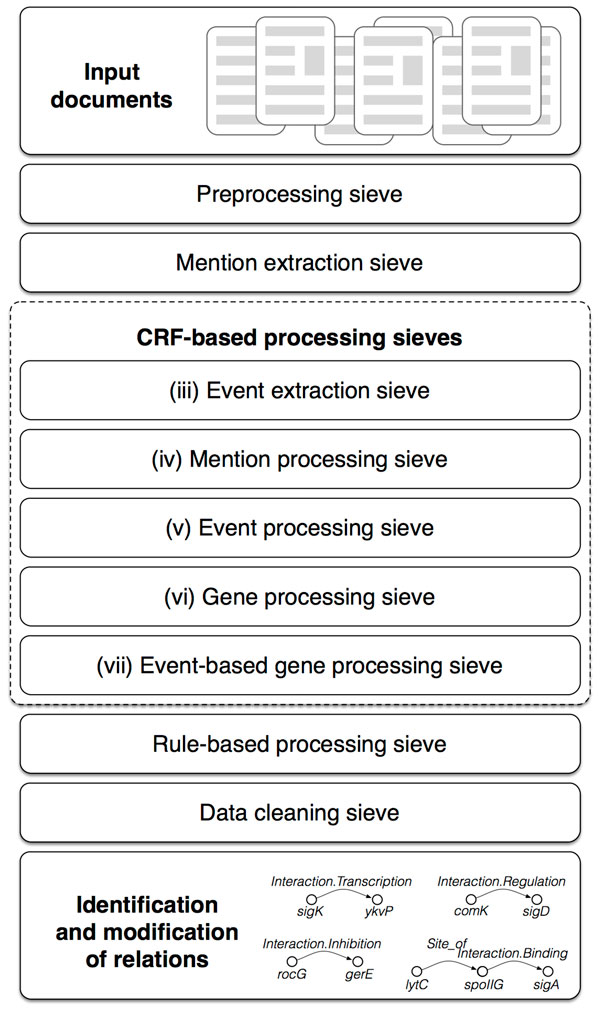
**Architecture of the proposed sieve-based relation extraction system**. The system consists of nine sieves. The first two prepare data for processing, then six sieves try to recognize events and relations, and the last sieve cleans the extracted relations. Every input document is processed sequentially by each of the sieves and at the end a list of extracted relations is returned as a result.

This paper aims at the extraction of gene regulatory networks of *Bacillus subtilis*. The reconstruction and elucidation of gene regulation networks is an important task that can change our understanding of the processes and molecular interactions within the cell [17-19]. We have developed a novel sieve-based computational methodology that builds upon conditional random fields [20] and specialized rules to extract gene relations from unstructured text. Extracted relations are assembled into a multi-relational gene network that is informative of the type of regulation between pairs of genes and the directionality of their action. The proposed approach can consider biological literature on gene interactions from multiple data sources. The main novelty of our work here is the construction of a sequential analysis pipeline for extracting gene relations of various types from literature data (Figure 1). We demonstrate the effectiveness and applicability of our recently proposed coreference resolution system [21]. Our system uses linear-chain conditional random fields in an innovative way and can detect distant coreferent mentions in text using a novel transformation of data into skip-mention sequences.

We evaluate the proposed methodology by measuring the quality of extracted gene interactions that form the well studied regulatory network of sporulation in bacteria *B. subtilis*. Sporulation is an adaptive response of bacteria to scarce nutritional resources and involves differential development of two cells [22,23]. Many regulatory genes that control sporulation or direct structural and morphological changes that accompany this phenomenon have been characterized in the last decade [24,25]. The topology of bacterial sporulation network is stable and suffers no controversy; thus, it is appropriate to serve as a reference network against which the performance of relation extraction algorithms can be compared. Our evaluation demonstrates that the proposed approach substantially surpasses the accuracy of current state-of-the-art methods that were submitted to the Gene Regulation Network (GRN) BioNLP-ST 2013 Challenge (http://2013.bionlp-st.org/tasks/gene-regulation-network). The source code of our approach is freely available [26]. In this paper we represent a network extraction algorithm, which is an improvement on our winning submission to BioNLP 2013 [27]. With these improvements we have been able to further reduce the prediction error from 0.73 to 0.68, measured as the slot error rate (SER). This paper substantially extends our previous work [27]. Below, we discuss motivation for using skip-mention sequences by analyzing distributions of distances between various parts of text (i.e., mentions) that are used by specialized sieves. We further explain feature functions and rules as they are key components of the system. We analyze the number of relations extracted by each sieve. The approach described here adds a new conditional random fields (CRFs) sieve to detect direct relations between *B. subtilis *genes that are "hidden" as target mentions within events. To better address text from biomedicine, we use the BioLemmatizer [28] instead of a general lemmatizer. We incorporate an additional knowledge resource - *B. subtilis *protein-protein interaction network from the STRING database [29], which is used within the new feature function *BSubtilisPPI*.

We use the term sieve to represent a separate relationship processing component. As we may extract new relationships or delete them in each of the sieve, the term might not be well selected but we left the terminology to comply with the previously published conference paper [27] and the coreference resolution system [30] that inspired the architecture of our proposed system.

### Related work

Research in the field of relationship extraction focuses on extraction of binary relationships between two arguments. New systems are typically tested using social relationships in the Automatic Content Extraction (ACE) evaluation datasets [31,32], where the goal is to select pairs of arguments and assign them a relationship type. Machine learning approaches that have been used for relationship extraction include sequence classifiers, such as hidden Markov models [33], conditional random fields [20], maximum-entropy Markov models [34] and binary classifiers. The latter usually employs support vector machines (SVM) [35].

The ACE 2004 dataset [36] consists of two-level hierarchical relationship types. A relationship could have another relationship as an argument and a second level relationship can have only non-relationship-like arguments. Two-level relationship hierarchies could have a maximum tree height of two. Wang *et al*. [32] proposed a system that uses a one-against-one SVM classifier to classify relationships in the ACE 2004 dataset by employing WordNet [37]-based semantic features. The GRN BioNLP 2013 Shared Task aimed to detect three-level hierarchical relationships. These relationships are interactions that connect events or other types of interactions as arguments. In comparison to the pairwise technique [32], we extract relationships using linear-based sequence models and manually defined rules.

A relation could be written using forms in unstructured text. Machine learning techniques try to learn diverse relations by adapting models against large datasets and by exploiting informative text features. The features are instantiated by a predefined set of feature functions, which are applied on a specific dataset. A technique to overcome a low number of instances of diverse relationship forms was proposed by [38]. They proposed lexical-syntactic feature functions based on patterns that are able to identify dependency heads. The proposed solution was evaluated against two relationship types and two languages, where they achieved promising results. In this work we define manually assigned rules to overcome the heterogeneity of the relationship representation.

Text used for training a relationship extraction model is most often tagged using the IOB (inside-outside-beginning) notation [39]. In the IOB, the first occurrence of the relationship word is labeled as *B-REL*, second and later consecutive tokens, which also represent relationships are labeled as *I-REL*, and all other tokens are *O*. Part of the text that most closely identifies a known relationship between the two arguments is referred to as a relationship descriptor. Li *et al.*[40] used a linear-chain CRF model to label such descriptors. They first changed the subject and object arguments of the accompanying relationships into a specific value (e.g., ARG-1, ARG-2). This transformation enabled them to correctly identify direction of a relationship. Moreover, they also merged all the tokens from a relationship descriptor into a single token, which enabled them to use long distance features using a linear model representation. We employ an analogous model representation, but transform a sequence of tokens in an innovative way that enables us to extract the target relationship type between the arguments and not just a relationship descriptor. Banko and Etzioni [41] also employed linear-based classifiers for the *open *relationship extraction problem, that is, the identification of a general relationship descriptor without regard to any target relationship type. First, they analyzed specific relationship types in the text taking into account lexical and syntactic features and then they learned a CRF model against with synonym identification [42]. Their approach is useful in scenarios where only a very limited number of relationships are known. Traditional relationship extraction methods can perform better if our goal is a high value of recall. For this reason we focus on supervised relationship extraction model.

Relationship extraction methods in biomedicine have been evaluated at several shared task challenges. The LLL - Learning Language in Logic challenge on gene interaction extraction [43] is related to the BioNLP 2013 Gene Regulatory Networks Shared Task, which includes a subset of the LLL data with some additional annotations. For the LLL task, Giuliano *et al*. [44] used a SVM classifier and proposed a specialized local and global SVM kernel that uses neighboring words as contextual information. The local kernel was based solely on mention features, such as words, lemmas or part-of-speech (POS) tags. In contrast, the global kernel used tokens on the left side of, between and on the right side of pairs of mentions that represent candidate arguments. To identify relationships, Giuliano *et al*. processed documents that contained at least two candidate attributes and generated $\left(\begin{array}{c} n\\ k \end{array}\right)$ example instances, where *n *was the number of all mentions in a document and *k *was the number of mentions that constituted a relationship (i.e., two). Giuliano *et al*. used their model to predict either a non-existing relationship, a subject-object relationship or an object-subject relationship. On a related note, we propose the usage of contextual features and syntactic features that depend on neighboring words. However, we predict unoriented extracted relationships and then determine their directionality, i.e., the subject and object arguments, through manually defined rules.

### Survey of BioNLP shared tasks

The BioNLP Shared Task challenges follow an established research-wide trend in biomedical data mining towards the specific information extraction tasks. Challenge events have been organized thus far in 2009 [45], 2011 [46] and 2013 [47-49], each co-located with the BioNLP workshop at the Association for Computational Linguistics (ACL) Conference. The first event triggered active research in the biomedical community on various information extraction tasks. Second shared task focused on generalizing text types and domains, and on supporting different event types. The most recent shared task took a step further and addressed the information extraction problems in semantic web, pathways, cancer-related molecular mechanisms, gene regulation networks and ontology populations.

The BioNLP 2011 Entity Relations challenge focused on the entity relationship extraction. The best performing system, called TEES [35], used a pipeline with SVMs for the detection of entity nodes and relation prediction that was followed by post-processing routines. It predicted relationships between every two candidate mentions within a sentence. The evalution showed that the term identification step could strongly impact on the performance of the relationship extraction module. In our case, proteins and mentions of entities, these are mentions that represent genes, were identified prior to the beginning of the challenge, and thus, our work here focused on the extraction of events, relations and event modification mentions.

In this work we describe the method that we developed while participating in the BioNLP 2013 Gene Regulation Network Shared Task [47]. We report on several refinements of our approach that were introduced after the shared task ended and that allowed us to further improve its predictive performance. The goal of the GRN task was to extract gene interactions from research abstracts and to assemble a gene network, which was informative of gene regulation. Training data contained manually labeled texts obtained from research articles that contained entity mentions, events and interactions between genes. Entities were text sequences that identified entities, such as genes, proteins or regulons. Events and relationships were defined by their type, two connected arguments (i.e., entities) and the direction between the arguments. Given a test dataset, our goal was to predict relations describing various types of gene interactions. Predicted network of extracted gene interactions was matched with the reference gene regulatory network and scored using a Slot Error Rate (SER) [50]. The SER measures the proportion of incorrect predictions relative to the number of reference relations.

## Methods

In this section we present our proposed sieve-based system for relation extraction. We start by describing the linear-chain conditional random field (CRF) model and proceed by extending it with a novel data representation that relies on skip-mentions. We provide support for transforming data into skip-mention sequences by studying various mention distributions that are used by CRF-based sieves. We then overview feature functions used by our model and explain the sieve-based system architecture, which is an end-to-end procedure that consists of data preprocessing, linear-chain CRF execution, rule-based relationship identification and data cleaning.

### Conditional random fields with skip-mentions

CRF [20] is a discriminative model, which estimates distribution of the objective sequence **y **conditioned on the input sequence **x**, that is, *p*(**y***|***x**). Following is an example of the input sequence from the GRN BioNLP 2013 training dataset, where the potential attributes (i.e., mentions) are shown in bold:

"**spo0H **RNA and **sigma H levels **during growth are not identical to each other or to the pattern of **expression **of **spoVG**, a gene **transcribed **by **E sigma H**."

The corresponding objective sequence for this example is **y **- [*O, O, EVENT, O, EVENT, O, TranscriptionBy*], which also corresponds to tokens in **x **- [*spo0H, sigma H, levels, expression, spoVG, transcribed, E sigma H*]. Thus, both sequences are of the same length.

**Figure 2:**
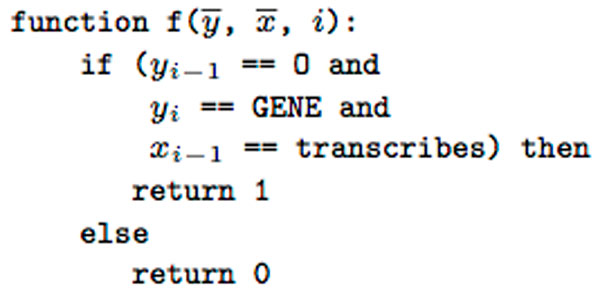
**A feature function example**. The feature function indicates whether the current label is *Gene*, the previous is *Other *and the previous word is "*transcribes*", which returns 1 or otherwise it returns 0.

We retrieve additional information for input sequence **x **and generate sequences **x**^LEMMA^, **x**^PARSE^, **x**^POS ^that contain lemmas, parse trees, tokens and part-of-speech tags for each corresponding token in **x**. The CRF considers feature functions *f_j_*, where *j *denotes *j*-th feature function, *j *= 1, 2*, . . . , m *(Figure 2). Feature functions employ text sequences to model target sequence **y**. The design of appropriate feature functions is the most important step in training CRF models. They contribute substantially to the improved performance of the system. We implement feature functions as templates and generate the final feature set by evaluating feature functions on a training dataset. The feature functions used by our model are described in the following section.

Training of a CRF model involves estimating the most probable objective sequence $\hat{y}$ˆ given the input **x**. In particular, we estimate

$$\hat{y}=\underset{\text{y}}{\text{argmax}\;p(\text{y|x},\;\text{w}),}$$

where **w **is a vector of model parameters, weights, that have to be learned. Here, the conditional distribution *p*(**y***|***x**, **w**) is written as

$$p\left(\text{y|x},\;\text{w}\right)=\frac{\text{exp}\left(\sum_{j=1}^{m}w_{j}\sum_{i=1}^{m}f_{j}\left(y,\;x,\;i\right)\right)}{C\left(\text{x,}\;\text{w}\right)},$$

where *n *represents the length of input sequence **x**, *m *the number of feature functions and *C*(**x**, **w**) is a normalization constant over all possible objective sequences **y**. Here, *f_j _*(**y**, **x***, i*) denotes a *j*-th feature that is fired for *i*-th place in the input sequence. In our computations we avoid the need of computing normalization constant *C*. Instead of using the exact probabilities we rather rely on ranking of the sequences relative to their probabilities and return a sequence that is ranked first. use features that are fired at least five times on the training data (a parameter to our system).

**Figure 3:**
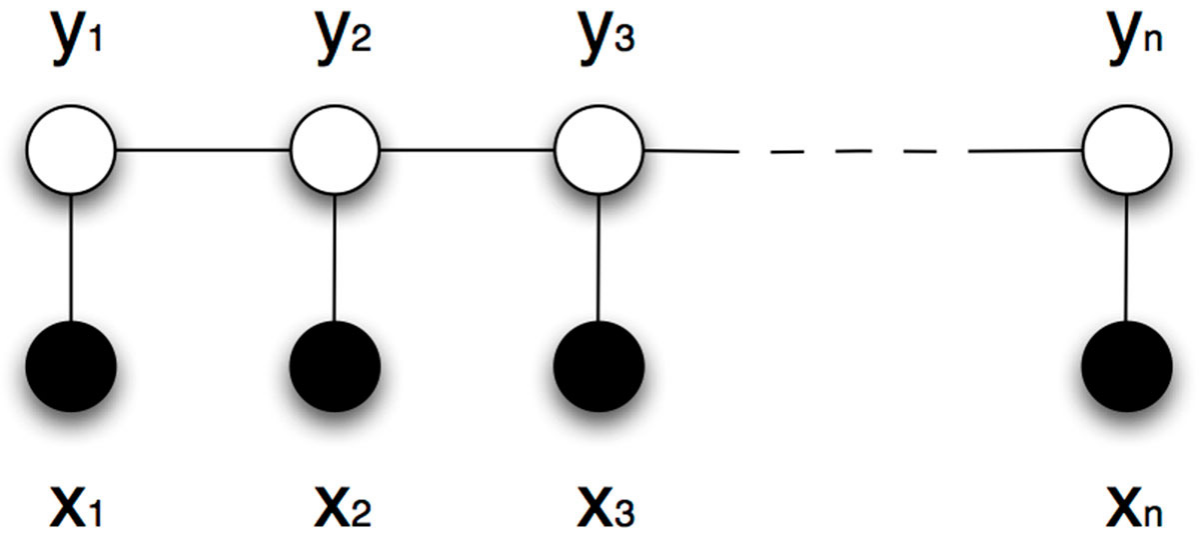
**The linear-chain conditional random fields model representation**. The model is represented with an input sequence **x **(e.g., words) and target sequence **y **(i.e., relationship names) containing *n *tokens.

The structure of a linear-chain model depends on the references to the target sequence labels that are used by the input feature functions. Figure 3 shows the graphical representation of the linear-chain CRF model. From the figure we can observe that the *i*-th factor can depend only on the current **y***_i _*label and the previous label **y***_*i−*1 _*in a sequence. The training of linear CRFs is fast and efficient. This is in contrast to more complex CRF models, whose model inference is in general intractable and requires approximate probabilistic methods.

#### Model definition

**Figure 4:**
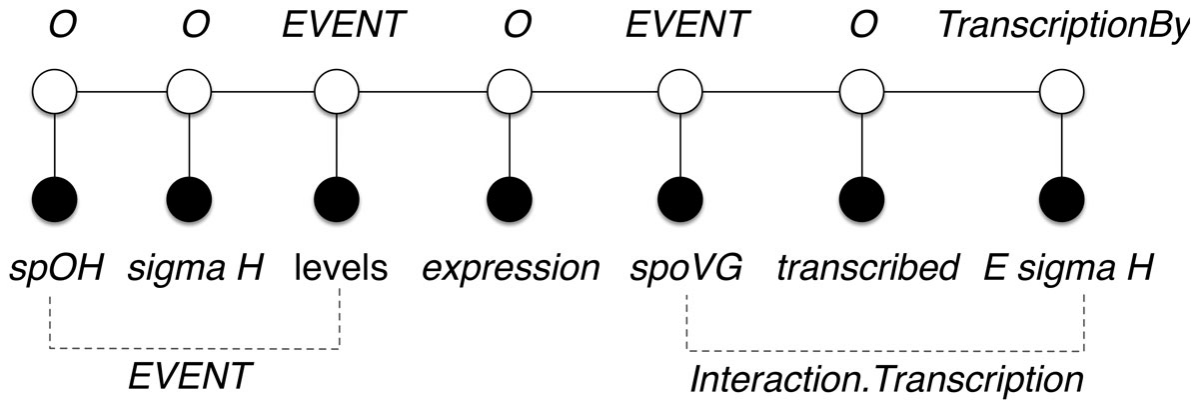
**Zero skip-mention sequence**. The initial mention sequence that contains all the mentions (i.e., zero skip-mention) from the document "spo0H RNA and sigma H levels during growth are not identical to each other or to the pattern of expression of spoVG, a gene transcribed by E sigma H."^1 ^A sentence from the GRN BioNLP 2013 training dataset, article PMID-1898930-S9.

We formulate the task of relationship extraction as identification of relationships between two arguments. Linear-chain CRF model with standard data representation lacks the modeling of dependencies between mentions on longer distances (i.e., arguments that have at least one other token in-between). By analyzing the example from the previous section, "gene **transcribed **by **E sigma H**", we conclude that untransformed data representation can only identify relationships between two consecutive tokens. Thus, we cannot extract all possible relationships using a linear model. Rather than extracting relationship descriptors (i.e., parts of text that identify a relationship), we would like to extract categorized relationships between pairs of mentions. To overcome the limitation of linear models, we introduce new sequences that contain only mentions. We refer to these sequences as mention sequences. Mentions are a type of arguments that can form a relationship. In Figure 4 we present a conversion of the text excerpt into a mention sequence. Transformed sequence **x **consists of consecutive entity mentions. Notice that entity mentions are included in the training dataset.

We label target sequence **y **with the name of a relationship (e.g., *Interaction.Transcription, EVENT*) or with the none symbol (i.e., *O*) when no relationship is present. Each relationship label represents a relationship between the current and the previous mention.

**Figure 5:**
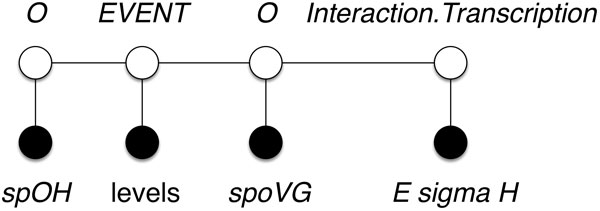
**One skip-mention sequence**. One out of two possible one skip-mention sequences, generated from the initial zero skip-mention sequence [*spOH, sigma H, levels, expression, spoVG, transcribed, E sigma H*]. The other one consists of tokens *sigma H, expression *and *transcribed*.

From the mention sequence generated in Figure 4, we cannot identify relationships between mentions that are not consecutive. This limitation becomes exacerbated when mentions that are arguments of a certain relationship appear on longer distances. For example, mentions *spoVG *and *E sigma H *should be related via the *Interaction.Transcription *relationship. However, this relationship cannot be extracted from representation that considers only consecutive mention pairs. Furthermore, a linear model can only detect relationships between directly consecutive mentions. To overcome this problem, we introduce a novel sequence representation called *skip-mention *sequences. The number of skip-mentions defines the number of mentions from the original text that exist between two consecutive mentions in a given skip-mention sequence. Thus, the original mention sequence (Figure 4) is a zero skip-mention sequence, because there are zero other mentions between any two consecutive mentions. This is opposed to a one skip-mention sequence, which considers relationships that are one mention apart. For example, to prepare the input data for extracting relationships between every second mention, we create two one skip-mention sequences for each input document. In the example in Figure 5 we extract relationship *Interaction.Transcription *based on one skip-mention sequence.

**Figure 6:**
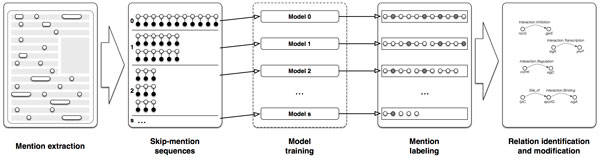
**Data flow in CRF-based relation extraction sieves**. First, the initial skip-mention sequence is transformed into the selected skip-mention sequences. Then, for each of the skip-mention sequence type, a different CRF model is trained and then used to label the appropriate skip-mention sequences. After labeling, the relations are instantiated from the tagged sequences and returned as a result.

**Figure 7:**
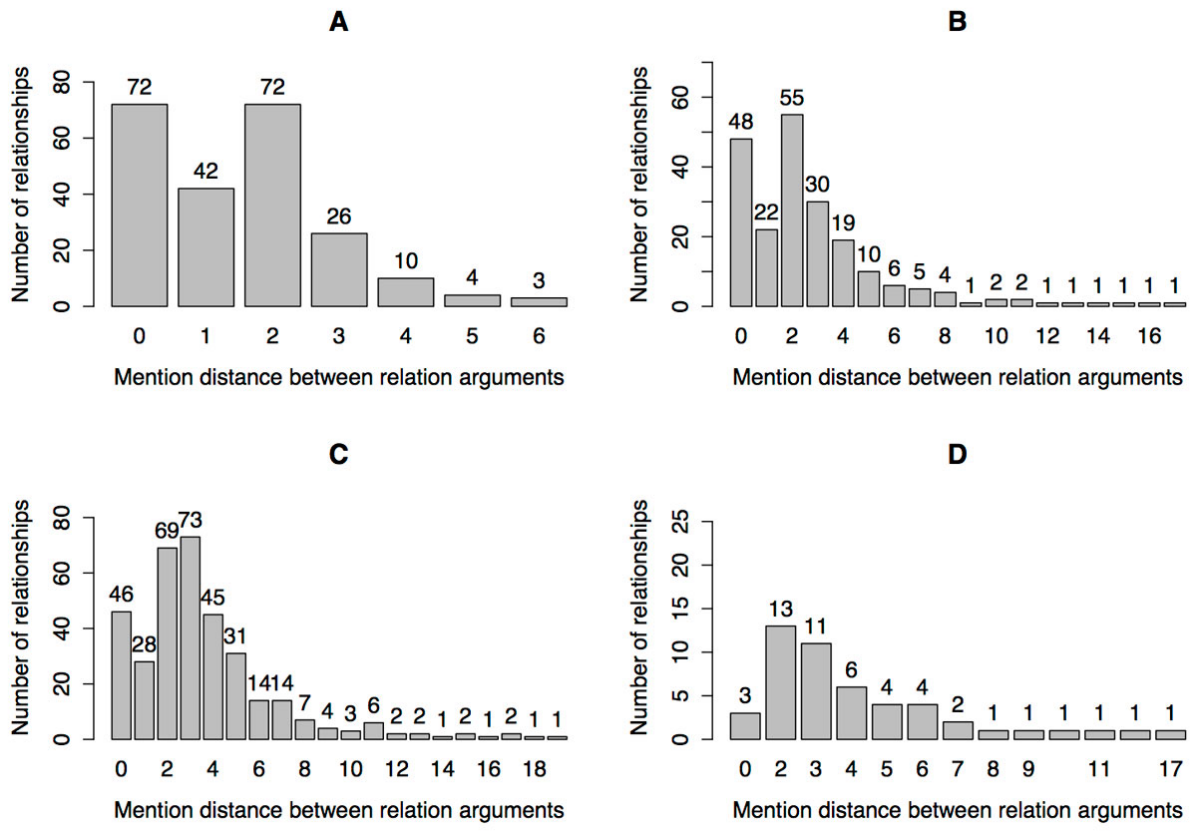
**Distributions of distances between relation attributes on BioNLP GRN train dataset**. (A) Mention distance distribution for events. (B) Mention distance distribution for relations. (C) Mention and event distance distributions for relations. Events are transformed into mentions. (D) A distribution of distances for relations in which subject and object mentions refer only to *B. subtilis *genes.

In a general setting we consider skip-mention sequences for mentions at distance *s*. For a given skip-mention number, *s*, we create *s *+ 1 mention sequences of length $\left[\frac{n}{s}\right]$. After the sequences are created, one independent linear-chain CRF model is trained for each value skip-mention number. As the generated sequences are independent, we can infer prediction models in parallel. From the models we read the extracted relationships between the mentions and form an undirected graph, where each connected component represents a relationship. Figure 6 shows a high level representation of data flow and relation extraction used in our approach. The time complexity of the proposed method is mainly determined by the time needed for training linear CRF models, since other routines can be run in linear time. Due to the parallel execution of the for loop (0, 1, 2*, . . . , s*), we need to find the longest lasting execution. Let us suppose that CRF training and inference has time complexity of *O*(*EL^Q^*) [51], where *E *is the number of edges in the graph, *L *is the number of labels, and *Q *is the size of the maximal clique. In our type of CRF model, we use one label for each relationship type. The number of edges *E *depends on the sequence input to the algorithm. Let further assume there are *n *mentions in a document, which results in a zero skip-mention sequence with 2*n − *1 = *O*(*n*) edges. Moreover, every other generated *s *skip-mention sequence contains $s\left(\left\lceil \frac{2n}{s}\right\rceil -1\right)=2n-s=O\left(n\right)$ edges. We conclude that by employing parallelization, CRF models would use *O*(*nL*^2^) = *O*(*n*) of time (number of labels *L *is small and fixed). In addition to other linear time procedures, it is also important to consider the time for initialization of feature functions, which takes on the order of *O*(*nm*), where *m *is the number of input feature functions. Figure 7 shows the distribution of distances between the relationship mention arguments (i.e., agents and targets) from the BioNLP 2013 Gene Regulatory Network training dataset. The labeled arguments represent entity mentions or events, depending on the sieve setting. Event is a type of relation that contains only mentions as their attributes. Events are extracted using the event extraction sieve. The distribution of distances between mentions is shown in the part A of Figure 7. In the sieve (iv) we identify relationships that have only mentions as their attributes (B). In the training data there are 153 relations that have another relation or an event as their attribute. Of these, there are 11 such relations that have another relation as their attribute. Seven contain a regular relation as an attribute, while four represent negated relations, which are not scored. Relations that contain events as attributes are extracted by the event relations processing sieve (v) and the distribution of distances between the attributes is shown in part C of the figure. To use the same approach as for the other sieves, we transform events into mentions (see the sieve (v) for details). Since hierarchies of events or relations are not considered in model evaluation, we include the gene relations processing sieve (vi). Sieve (vi) extracts relations only between mentions, that are identified as *B. subtilis *genes. The distribution of distances between such mentions is presented in part D in the figure. We notice a drop of number of relationships on distance one for parts A, B and C. This is due to the fact of all the mentions we take into account when forming mention sequences. Differently, in part D, we take only gene mentions into account which also results in not having a drop at distance one.

From all of the distance distributions we observe that relationships are mostly connected by the attributes on distance of two entity mentions. These distributions demonstrate the need to transform our data into skip-mention sequences. Without the transformation the linear-chain CRF model would, at best, uncover relations with attributes at zero distance (i.e., directly consecutive mentions).

For our final results we train the linear CRF models against skip-mention sequences from zero to ten skip-mentions. We decide to use this range after observing the distance distributions between attributes of the relations. By using up to ten skip-mentions we can retrieve most of relations and do not overfit the model. The findings in our previous work [21] show that after reaching the tail of distance distributions the results do not further improve.

The feature functions that we consider are thoroughly explained in Table 1 and Table 2. The tables contain short descriptions of the functions and parameters that are used for their instantiation. Additionally, the feature function generators generate a number of different functions from the training data and for them we also include the label types from which they are generated.

**Table 1:** Feature functions description.

Name	Description	Options
Target label distribution	Distribution of target labels.	--
Starts upper	Does a mention start with an upper case leter.	current, previous mention
Starts upper twice	Do two consequent mentions start with an upper case letter.	current, previous mention
Hearst co-occurence [58]	Does the text between the two mentions follow some predefined rules, e.g., *mi *such as *mj*.	--
Mention token distance	Distance between the two mentions in number of mentions.	--
Parse tree mention depth	Depth of the mention within the parse tree.	--
Parse tree parent value	Parse tree value of the mention on length *l*	*l ∈ *{1, 2, 3}
Parse tree path	Path values between the two mentions in a parse tree, e.g., *DT/NP/NNS/.../NP/NP/VBG*.	up to three tokens from every mention
BSubtilis	If the two mentions are known as *B. subtilis*, what is the probability of protein-protein interaction using STRING data [29], i.e., very low, low, medium, high, very high.	--
IsBSubtilis	Is the current mention known as *B. subtilis *gene.	--
IsBsubtilisPair	Which of the two consequent mentions is known as *B. subtilis *genes, i.e., left, right, both or none.	--

The feature functions are used by all CRF-based sieves for all selected skip-mention CRF models. All extracted features are modeled both as unigram and bigram features. Unigram features are used for current label factor and bigram features are used for transition factor between two labels.

**Table 2:** Feature function generators description.

Name	Description	Options	Observable data
Prefix value	Value of the prefix for the mention on offset distance from the current mention.	string length: {2, 3}; offset: [−5, 5]	text
Suffix value	Value of the suffix for the mention on offset distance from the current mention.	string length: {2, 3}; offset: [−5, 5]	text
Consequent value	A combination of values of the two consequent mentions on offset distance from the current mention, *e.g., PDT/NNS*.	offset: [−4,4]	text, part-of-speech, lemma, entity type, coreference
Current value	A value of the mention on offset distance from the current mention, e.g., NNS.	offset: [−4,4]	text, part-of-speech, lemma, entity type, coreference
Context value	Matching of specified length of character-based ngram values within the selected range of words from the current and previous mentions using Jaccard coefficient. According to the match result, feature function values are discretized into eight levels. Different feature functions are generated for the context left/right of both mentions, between the two, outside the two and union of all.	range: 5, ngram: 3	text
Previous / next value combination	A combination of token values from the selected distance to the current and the previous mentions.	distance: {−2, 2}	text, part-of-speech, lemma
Left / right / between value	Token values on the left/right or in between the two mentions on the selected distance.	distance: [1,5]	text, part-of-speech, lemma
Split to values	Split the current mention into tokens by the selected delimiter and output first N tokens.	N: 2, delimiter: '	text, lemma

According to the implementation, different options and observable values, the generators generate specific feature functions using a single scan over training data. The feature functions are used by all CRF-based sieves for all selected skip-mention CRF models. All extracted features are modeled both as unigram and bigram features (except prefix and suffix, which are of unigram type only). Unigram features are used for current label factor and bigram features are used for transition factor between two labels.

### Data processing components

We introduce a pipeline-like data processing system that combines multiple data processing sieves (see Figure 1). Each is a separate data processing component. The whole system consists of nine sieves. The first two deal with data preprocessing and data preparation for efficient relationship extraction. The main ones then consist of linear CRF-based and rule-based relationship detection. The last one cleans the data before returning it as a result. The whole implementation of this proposed pipeline is available in a public source code repository [26]. CRFSuite [52] is used for fast CRF training and inference.

The proposed system can be easily adapted to another domain or other relation extraction task. In order to use it for other purposes, we would need to adapt the preprocessing part to enable the import of the new data. Also, the rule-based processing sieve would need to be discarded or populated with specific rules according to a new problem. All other sieves that extract relations could be the same because they use trained models and those would be specific to a domain and task. We also employed the use of skip-mention sequences to the task of coreference resolution and achieved comparable results to existing approaches [21]. The pipeline starts by transforming the input text into the internal data representation, which could be used for further processing and enriches the data with additional labels, such as part-of-speech tags, parse trees and lemmas. After that we detect also action mentions, which are attributes within events. Next, we employ linear CRF models for event detection. We represent events as a special relationship type. Then the main relationships processing sieves detect relationships. We propose several processing sieves for each of the relationship type based on the argument types or hierarchy support. After each relationship extraction step we also use rules to set the agent and target attributes in the right direction. The last relationship processing sieve performs rule-based relationship extraction and therefore detects relationships of higher precision and boosts recall levels. In the last step the extracted data is cleaned and exported.

**Figure 8:**
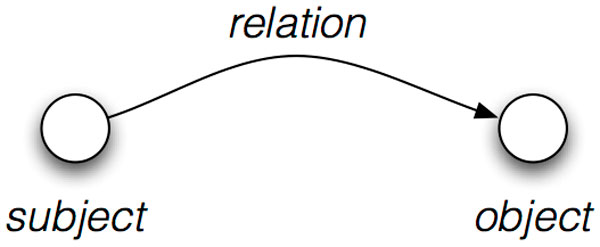
**General relation representation**. Each relation (e.g., gerE inhibits cotD) is defined with a name (e.g., *Interaction.Regulation*) and subject (e.g., *gerE*) and object (e.g., *cotD*) attributes.

The sieves of our system are run in the same order as shown in Figure 1. We provide detailed description of the processing sieves in the following sections, where we refer to the relationship attributes as subjects and objects, as shown in Figure 8. Notice that sieves can depend on each other if they use data extracted by sieves executed earlier in the system pipeline (i.e., sieve (iii) and (v)). The initial set of the mentions is produced by the mention extraction sieve. This set is then used throughout the system and represent relation attributes used by extracted relations.

#### Preprocessing sieve

Preprocessing phase includes data importation, detection of sentences and tokenization of input text. Additionally, we tag the data with new labels, which are lemmas [28], parse trees [53] and part-of-speech tags.

#### Mention extraction sieve

The entity mention can belong to any of the following types: *Protein, GeneFamily, ProteinFamily, ProteinComplex, PolymeraseComplex, Gene, Operon, mRNA, Site, Regulon *and *Promoter*. Entity mentions are provided with the corpus, however, action mentions (e.g., *expresses, transcribes*) are not included in the corpus. We automatically detect action mentions. They are needed to represent relationship arguments within events during the event extraction. To identify action mentions we gather action mention lemmas from the training dataset and select new candidate mentions from the test dataset by exact matching of the lemmas.

#### Event extraction sieve (iii)

An event can be defined as a change in the state of biological entities, such as genes or complexes (e.g., "the pattern of *expression *of *spoVG*"). We encode events as a special relationship with a type name "*EVENT*". In the dataset, the event subject types can be of *Protein, GeneFamily, PolymeraseComplex, Gene, Operon, mRNA, Site, Regulon *and *Promoter *types, while the objects are always of the action mention type (e.g., "*expression*"), which are discovered in the mention extraction sieve. After the event type relationships are identified, we employ manual rules that change the order of arguments - they set an action mention as the object and a gene as the subject attribute for all extracted events.

#### Relation processing sieves (iv, v, vi, vii)

Due to the existence of different relationships (i.e., different subject and object types), we extract relationships in four phases (iv, v, vi, vii). This also enables us to extract hierarchical relationships (i.e., relationships that contain another relationship as its subject or object) in order to achieve higher precision. All the sieves in this step use the novel linear CRF-based relationship extraction method. Each processing sieve uses specific relationship properties and is executed in the following order (the shown examples are sample extractions from the above demonstrative document):

(iv) First, we extract relationships with only mentions as arguments (e.g., *transcribed → TranscriptionBy → E sigma H*). Mentions can be either of the real or action type. By real mentions we refer to the entities that represent genes, proteins and aggregates, while action mentions could represent only arguments within events (e.g., transcription).

(v) In this step, we extract relationships that consist of at least one event in their arguments (e.g., *expression spoVG → Interaction.Transcription → E sigma H*). Before the extraction we map events into mentions, which enables us to use the same approach as in previous step. These mentions consist of two tokens (i.e., event arguments). We treat the newly created *event mentions *the same as others and also include them in the list of other mentions. Their order within the list is determined by the lowest mention token from the event. We train the models using the same techniques as in every other CRF-based processing sieve. The new action mentions are treated as other mentions and from them we extract features using the same set of feature functions. Lastly, the final relationships are instantiated following the same procedure as in the previous step.

(vi) The goal of the shared task is to extract *Interaction *relations between *B. subtilis *genes. Thus, we select only mentions that represent *B. subtilis *genes and train the algorithm to predict the appropriate *Interaction *relations (e.g., *spoVG → Interaction.Transcription → E sigma H *if there was no transcription event). For the mention selection step we exploit a public database of the *B. Subtilis *genes from the NCBI available at http://www.ncbi.nlm.nih.gov/nuccore/AL009126.

(vii) We propose this new processing sieve in addition to the previous sieves, which we previously introduced in the BioNLP challenge submission [27]. The goal of the challenge is to extract interactions between genes. When there exists a relationship between a gene *G*1 and and event *E*, the final result in a GRN networks looks exactly the same if our system extracts a relationship between a gene *G*1 and a gene *G*2, where *G*2 is the object attribute of the event *E*. By taking into account the latter, we train the models to extract relationships only between *B. subtilis *genes (e.g., *spoVG → Interaction.Transcription → E sigma H*, where *spoVG *is the subject attribute within an event).

The challenge datasets include seven hierarchical relationship instances, which have another relationship as one of its arguments. Due to the small number of instances and newly introduced relationship extraction sieve between genes (vi, vii), we did not extract this type of relationship hierarchies.

Additionally, there exist four negated relation instances. The BioNLP task considers only positive relations and there is no performance gain if negated relations are extracted. Thus, we focus on extracting positive relations. Depending on the dataset and performance evaluation measure, we can add a separate sieve that can extract negated relations by applying manually defined rules that search for negation words such as *nor, neither, whereas *and *not*.

#### Rule-based processing sieve

The last phase of relationship extraction involves application of the rules to achieve higher precision. The rules operate directly on the input text with recognized mentions and use different data representation than extractors based on CRFs. We implemented the following four approaches:

**Mention triplets: **This method searches for the consequent triplets of mentions, where the middle mention is an action mention. As input to the rule we set the matching regular expression that searches for text that action mention must starts with, and a target relation. For example, from text "The *rocG *gene of Bacillus subtilis, encoding a catabolic glutamate dehydrogenase, is *transcribed *by *SigL *. . . ", we extract a relation *rocG → Interaction.Transcription → SigL*. The mention triplet in this example is *rocG, transcribed *and *SigL*, where the middle mention is an action mention matching the regular expression.

**Consecutive mentions: **The method processes every two consequent *B. subtilis *entity mentions and checks whether the text in-between the mentions matches a specified regular expression used for extracting a target relation. By default, it forms relations that are extracted from active sentences, otherwise it supposes the passive type and changes the order of attribute types within the matched relation. For example, from text "*GerE *binds to a site on one of these promoters, *cotX*, that. . . ", we extract relation *GerE → Interaction.Requirement → cotX*. Notice that mentions *GerE *and *cotX *represent the *B. subtilis *entities and text between the entities matches a regular expression ".*binds to.*".

**List of consecutive mentions: **This method extends the technique designed for consecutive mentions by allowing potentially many entity mentions on both sides of matched regular expression. The list of mentions must be separated by one of the delimiters ",", ", and" or "and". For example, this rule extracts two relationships from the sentence "the *cotG *promoter is induced under the control of the *sigma K *and the DNA-binding protein *GerE*."

**Sentences of consecutive mentions: **This method is similar to the rule for consecutive mentions. It first removes subsentences that exist between two mentions and then it extracts relationships. Subsentences are defined as parts of text between two commas. For example, the method extracts a relationship *GerR → Interaction.Requirement → SpoIIID *from the sentence "The *sigma(E) *factor turns on 262 genes, including those for *GerR*, and *SpoIIID*.".

The *Interaction *relationships are extracted using keywords and regular expressions that depend on the type of interaction. Biomedical literature uses many different language forms to express the same type of a genetic relationship. For example, some researchers prefer *to repress *to *to inactivate *or *to inhibit*. We use synonyms of this kind to extract additional relationships that are not identified by linear CRF models. The parameters used for rule-based extraction are shown in Table 3.

**Table 3:** Rule-based processing sieve input parameters.

Regular expression	
**Mention triplets** **Transcription**	**transcrib**
Consecutive mentions	
Transcription	.*directs transcription.*
Inhibition	.*inactivate.*
Inhibition	.*inhibits.*
Inhibition	.*repressor to.*
Inhibition ^1^	.*is negatively regulated by.*
Activation^1^	.*is governed by.*
Activation^1^	.*essential.*activat.*
Activation	.*to.*activat.*
Activation	.*turns on.*
Requirement^1^	.*requires.*
Requirement	.*required.*
Binding	.*binds.*to.*
Binding	-binding.*
List of consecutive mentions	
Transcription	.*under.*control.*of.*
Activation^1^	.*is governed by.*
Inhibition	.*represses.*
Inhibition	.*to repress.*
Sentences of consecutive mentions	
Activation	.*turns on.*
Inhibition	.*repressed.*

Each of the four different rule-based extraction methods takes a target relation name and a regular expression as input. Some of them also require to specify whether the extraction should be made from active or passive sentences.

^1 ^The method is called with passive parameter set to true.

#### Data cleaning sieve

The data cleaning sieve removes loops of relationships and eliminates redundancies. We call relationship a loop if and only if both relationship arguments refer to the same entity (i.e., mentions are coreferent). For example, the sentence "... *sp0H *RNA and *sigma H *..." refers to the mentions *sp0H *and *sigma H*. Since both mentions refer to the same entity (i.e., *sigH*), they cannot form a relationship. Removal of the loops improves performance of the system as it contributes to the reduction of undesired insertions in the final prediction. Another step in data cleaning phase is removal of redundant relationships. Disregarding redundant relationships has no affect on predictive performance of our system but it improves the readability of the output.

## Experimental setup

### BioNLP GRN 2013 challenge dataset

The GRN dataset consists of sentences from PubMed abstracts, which are mostly related to the topic of sporulation in *B. subtilis *and from which an appropriate gene regulation network can be reconstructed. It contains annotated text-bound entities that we call mentions. These mentions include biochemical events and relationships that were result of already conducted research work on cellular mechanisms at the molecular level. The goal of BioNLP Shared Task was to identify interactions, which represent relations between biological entities, events or relations and are essential for construction of GRN. The interaction relations form a hierarchy of mechanism and effect relation types. We were required to predict the following fine-grained interaction relation classes: *regulation, inhibition, activation, requirement, binding *and *transcription*.

In Table 4 we report on the features of the train, development and test datasets that were used in our study. The test dataset does not include labeled data and thus we cannot perform the evaluation of each sieve against it. In the other two datasets the sentences are manually labeled with relationships, events and entity mentions.

**Table 4:** BioNLP 2013 GRN Shared Task development, training and test dataset properties.

Dataset	dev	train	test
Documents	48	86	67
Tokens	1321	2380	1874
Real mentions	205	422	290
Action mentions	55	102	86
Events	72	157	--
Relations	105	254	--
Interaction relations	71	159	--

The numbers of the Interaction relations that our system reads from the datasets is different than the real ones due to the import technique into our internal data representation. The dev dataset contains 67 and training dataset contains 131 reference Interaction relations. The test data contains 88 such relation instances (The number was retrieved from the output of the official BioNLP GRN Shared Task test evaluation service).

### Evaluation criterion

The official evaluation criterion of the BioNLP challenge considers edge resemblance between the predicted and the reference gene regulatory network describing sporulation in *B. subtilis*. The performance of a relation extraction system is evaluated using the SER measure [50]

$$SER=\left(S+I+D\right)/N,$$

which is the ratio between the sum of relationship substitutions (S), insertions (I) and deletions (D), divided by the number of edges in the reference network (N). In short, systems that output as many wrong predictions as correct predictions achieve a SER value of 1. Notice that a system, which reports zero extracted relations, produces as many deletions as there are relations in a dataset (i.e., *N *= *D*). When a system extracts a true relation, the number of deletions decreases by one. If it detects a false relation then either the number of substitutions or the number of insertions increases by one. More accurate systems have a lower SER. A perfect system would correctly identify all relations and would achieve a SER of 0. Our goal is to maximize the number of matched relations and minimize the number of substitutions, deletions and insertions.

## Results and discussion

We represent the GRN relationship extraction challenge as a two-level task. First, we need to identify relationships among given labeled mentions and secondly, we need to correctly identify the argument types of extracted relationships (i.e., the direction of a relationship). For the challenge evaluation procedure, only results that match by relationship type and also by both argument types are counted as correct.

Our approach consists of multiple submodules, i.e., sieves, whereas each is developed for extracting a specific relationship type (e.g., are both arguments mentions, are arguments an event and a mention, or are both of them gene mentions). For the CRF-based relation extraction sieves we use skip-mention distances from zero to ten. Thus, we first show the overall results and then discuss the contributions of each sieve and subsets of feature functions.

### Predictive performance

We evaluated the proposed solution against the GRN BioNLP 2013 Shared Task dataset using leave one out cross validation on the development data, where we achieved a SER score of 0.74, with no substitutions, 36 deletions, 14 insertions and 31 matches. According to the results reported on the development dataset at the BioNLP workshop [27], this is improvement for one point in SER due to the additional sieve and new feature functions.

The challenge test dataset consists of 290 mentions from 67 sentences. We trained the models jointly on the development and train datasets to detect relationships against the test data. The challenge submission results of other participants in the shared task are listed in Table 5. According to the official SER measure, our system (U. of Ljubljana) was ranked first. The other participants or participating systems were K. U. Leuven [54], TEES-2.1 [55], IRISA-TexMex [56] and

**Table 5:** BioNLP 2013 GRN Shared Task results on the test dataset.

Participant	S	D	I	M	SER
U. of Ljubljana	8	50	6	30	0.73
K. U. Leuven	15	53	5	20	0.83
TEES-2.1	9	59	8	20	0.86
IRISA-TexMex	27	25	28	36	0.91
EVEX	10	67	4	11	0.92

The table shows the number of substitutions (S), deletions (D), insertions (I), matches (M) and slot error rate (SER) metric. Best results per metric are highlighted in bold. Reported are results announced after the BioNLP 2013 GRN challenge was closed.

EVEX [57]. All the participants were trying to achieve a low number of substitutions, deletions and insertions, while trying to increase the number of matched relationships. We obtained the lowest number of substitutions and good results in the other three counters, which resulted in the best SER score. In general also other participants generated a high number of deletions, which is a clear result that the relationships are encoded in many and ambiguous forms in the text. The IRISA-TexMex achieved the lowest number of deletions and the maximum number of matches but received a low final result due to a high number of insertions and substitutions.

Since the submission of our entry to the BioNLP challenge, we have introduced some new feature functions and implemented an additional sieve. The new sieve (vii) extracts relations between *B. subtilis *genes from hierarchically encoded relations in the training dataset. We report the improved results in Table 6. They all include new feature functions and are grouped by the inclusion of the new event-based gene processing (vii) sieve and data cleaning sieves. The result without both of them already outperforms our submitted result by one point, with a SER score of 0.72. The new feature functions extract more relations with increased precision. It is interesting that the inclusion of the sieve (vii) deteriorates the final result by about 4 SER points. However, the inclusion uncovers more matches, but it inserts a substantial number of non-correct relations, which results in a higher error rate. Thus, the best SER score of 0.68 was achieved without the sieve (vii) and with data cleaning. Compared to our winning result at the BioNLP Shared Task, this may further improve the system by 5 SER points.

**Table 6:** Results on test data.

Setting	S	D	I	M	SER
wo. (vii) & wo. cleaning	4	51	8	33	0.72
wo. (vii) & cleaning	4	51	5	33	0.68
(vii) & wo. cleaning	5	47	15	36	0.76
(vii) & cleaning	5	47	12	36	0.73

The table shows results on the test set using new feature functions and the additional sieve (vii) with or without data cleaning. The abbreviations represent the number of substitutions (S), deletions (D), insertions (I) and matches (M). Best results per metric are highlighted in bold.

**Figure 9:**
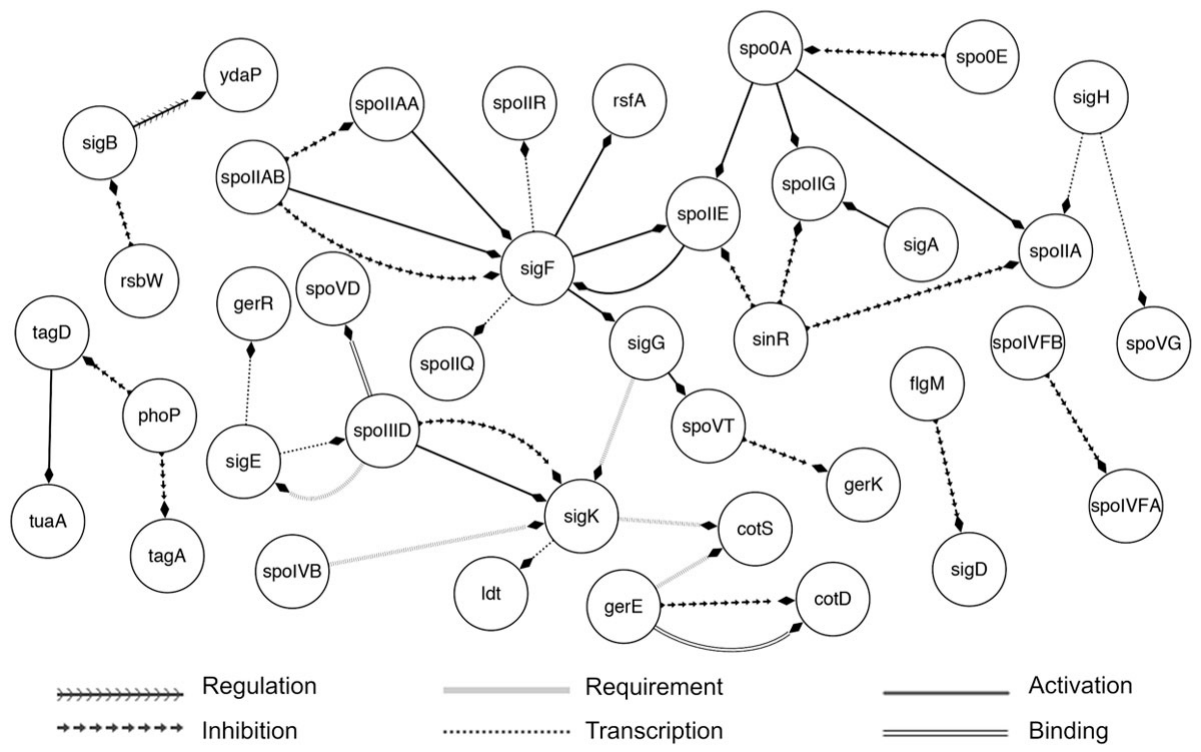
**Predicted gene regulation network on test data**. The predicted gene regulation network, generated from extracted relations on the test dataset by our improved sieve-based system. For our winning extractions at the BioNLP 2013 GRN Shared Task see the workshop paper [27].

In Figure 9 we show the gene regulation network, which is the visual representation of the results of our system against the test dataset. Compared to our shared task submission [27], the improved system identifies two additional relations (i.e., *spoIVFB → Inhibition → spoIVFA, sigE → Transcription → gerR*) and deletes one (i.e., *sigB → Transcription → yvyD*). If the deleted relation is correct, we could merge the results and achieve a SER of 0.67 with 4 substitutions, 50 deletions, 5 insertions and 34 matches, given 88 relations in the test set. To the best of our knowledge, this result represents the most accurate prediction on BioNLP GRN dataset so far. We were able to retrieve 39% of interactions from the data, which suggests that automatic extraction of gene regulatory networks is still a challenging task with open opportunity for future research.

### Analysis of extractions per sieve

Table 7 shows the number of extracted relations by each sieve. The same relation can be extracted by multiple sieves. Thus, we apply data cleaning as the last sieve to remove loop and duplicate relations.

**Table 7:** Relations extracted by each sieve on development and test datasets.

	Dev		Test	
**Sieve**	**#**	**SER**	**#**	**SER**
Event extraction	29	1.00	32	1.00
Mention processing	44	0.87	12	1.00
Event processing	11	0.84	2	1.00
Gene processing	14	0.84	5	0.97
Event-based gene processing^1^	26	0.73	15	0.96
Rule-based processing	12	0.75	53	0.76
Data cleaning	22/20	0.75	14/5	0.73

Data cleaning results represent the number of loop relations and the number of redundant relations (separated by forward slash). Slot error rate (SER) results are cumulative.

^1 ^Due to additional analysis we saw that the event-based gene processing sieve does not improve the final results, therefore we do not employ this sieve on the test data for the final result.

The event extraction sieve uncovers events, which we represent as relations. Events are not part of performance evaluation and thus their extraction does not directly affect the SER score. Extracted events are given as input to the event processing sieve, which extracts relations having an event as a relation attribute. The first two relation processing sieves (Figure 1) already achieve promising performance on the development dataset, while on the test set they extract seven correct and seven incorrect relations, that is, the SER score remains 1. The next two sieves extract more correct relations on the test set and achieve very good results on the development dataset. The event-based gene processing sieve shows substantial improvements on the development dataset, while there is a minor result change on the test set. The lowest SER score is achieved when not using this sieve for the test set (but the CRF models are trained on both training and development data). In this setting there are no further improvements when using rules on the development data. Notice that the rule-based sieve contributed importantly on the development data before we introduced the event-based gene processing sieve into the system. We observed that many relations previously extracted by rules are now detected by the event-based gene processing sieve. Contrary to development data, rules uncover substantially more relations on the test dataset than event-based sieves.

### Assessment of subsets of feature functions

The selection of the most informative feature functions is one of the key tasks in machine learning for improving the quality of results. In Table 8 we show the results on the development data when using different subsets of feature functions. Feature functions were grouped into subsets, ranging from more general (A-C) to more specific (D-H). As expected, the results improve when more feature functions are used. If only basic features (A) are applied, the system detects one wrong relation, which results in a SER higher than 1. Still, when using *B. subtilis*-related feature functions (C), the results show no improvement (Table 8). We notice a reduction of 0.12 in error rate when prefix and suffix feature functions (D) were added. Thus, we suspect that the improvement results from combining these functions with other feature functions (D) or it is due to D being generator feature functions that generate larger number of features than the previous (A-C) ones. Also, the next generator of mention values and mention pairs (E) substantially improves the result. This is expected, especially if the same type of relations exist in the development dataset and in the training dataset. We confirmed that D and E perform poorly if used separately, achieving a SER of 0.98 and 0.87, respectively. If D and E are used together, the system achieves a SER of 0.81. Thus, the inclusion of diverse feature functions is important. It may seem that the feature function subset H does not contribute to the results. This does not hold and can be seen if subset G is excluded. The latter configuration gives a SER of 0.74.

**Table 8:** Relations extracted by different subsets of feature functions on a development dataset.

Subset of feature functions	S	D	Dev I	M	SER
A	0	67	1	0	1.01
A - B	1	64	2	2	1.00
A - C	1	64	2	2	1.00
A - D	0	52	7	15	0.88
A - E	0	41	12	26	0.79
A - F	1	38	12	28	0.76
A - G	0	37	12	30	0.73
A - H	0	37	12	30	0.73

The table shows the number of substitutions (S), deletions (D), insertions (I), matches (M) and slot error rate (SER) metric. The results are measured on the development dataset using CRF-based sieves only. Best results per metric are highlighted in bold. The feature function subsets are selected as follows: (A) target label distribution, starts upper, starts upper twice, Hearst co-occurence, mention token distance, (B) parse tree mention depth, parse tree parent value, parse tree path, (C) BSubtilis, IsBSubtilis, IsBSubtilisPair, (D) prefix value, suffix value, (E) consequent value, current value, (F) context value, (G) previous/next value combination, left/right/between value and (H) split to values. For their detailed descriptions see Table 1 and Table 2.

## Conclusions

We presented a sieve-based system for relationship extraction from textual data. The system uses linear-chain conditional random fields (CRFs) and manually defined extraction rules. To enable extraction of relationships between distant mentions we introduced *skip-mention *linear CRF, which extends the applicability of a linear CRF model. We form skip-mentions by constructing many sequences of mentions, which differ in the number of mentions we skip.

With a SER score of 0.73 our approach scored best among the GRN BioNLP-ST 2013 submissions, outperforming the second-best system by a large margin. We described here a number of improvements of our approach and demonstrated their utility that may be used to further improve the result (to 0.67 SER score). The CRF-based sieves in our approach are independent processing components and can be trained against an arbitrary data domain for which labeled data exists. We anticipate the utility of our approach in related data domains and for tasks with corpora.

## Competing interests

The authors declare that they have no competing interests.

## Authors' contributions

S.Z., M.Z., B.Z. and M.B. designed the experiments. S.Z. and M.Z. performed the experiments. S.Z., M.Z., B.Z. and M.B. wrote the main manuscript text. All authors read and approved the final manuscript.
